# Delayed Diagnosis of Small Bowel Perforation following Abdominal Liposuction

**DOI:** 10.70352/scrj.cr.26-0480

**Published:** 2026-10-23

**Authors:** Toshifumi Shinbo, Yasumasa Nakasha, Kaname Ishiguro

**Affiliations:** Department of Surgery, Noto General Hospital, Nanao, Ishikawa, Japan

**Keywords:** liposuction, intestinal perforation, abdominal abscess, visceral injury, delayed diagnosis, plastic surgery

## Abstract

**INTRODUCTION:**

Although abdominal liposuction is generally considered a safe and widely performed cosmetic procedure, rare but serious complications, including visceral injury, have been reported. We report a case of delayed diagnosis of small bowel perforation after abdominal liposuction, resulting in intra-abdominal abscess formation that required emergency surgery. This case highlights that bowel perforation may remain clinically silent during and immediately after liposuction, leading to delayed diagnosis.

**CASE PRESENTATION:**

A 55-year-old Japanese woman presented with abdominal pain following abdominal liposuction. The patient had a history of 2 cesarean sections and no other significant medical history. During the liposuction procedure, no abnormal vital signs or operative findings were observed. Mild abdominal pain was noted on POD 1 and was initially considered to be postoperative pain related to liposuction. However, the symptoms progressively worsened, prompting the patient to present to our hospital on POD 6. Physical examination revealed localized rebound tenderness in the left lower abdomen. CT revealed a localized fluid collection containing gas around an edematous segment of the small intestine, consistent with an intra-abdominal abscess secondary to small bowel perforation. Emergency surgery revealed a localized abscess and a solitary 3-mm ileal perforation. We performed abscess drainage and partial small bowel resection. Histopathological examination revealed granulation tissue formation and marked fibroblast proliferation around the perforation site, suggesting that the perforation had occurred several days earlier. No other pathological findings or foreign bodies were observed. Based on the clinical course and pathological findings, liposuction was considered the most plausible explanation for the small bowel perforation, although a direct causal relationship could not be definitively established. The patient’s postoperative course was uneventful.

**CONCLUSIONS:**

Although rare, abdominal liposuction can result in visceral injuries such as bowel perforation. Depending on the severity of the injury, diagnosis may be delayed, leading to severe or potentially fatal consequences. Surgeons should maintain a high index of suspicion for visceral injury when evaluating patients with abdominal pain following liposuction.

## Abbreviation

SpO_2_
: peripheral oxygen saturation

## INTRODUCTION

In recent years, the number of liposuction procedures performed in cosmetic surgery has increased worldwide.^1)^ Abdominal liposuction is a procedure in which small incisions are made in areas such as the umbilical region and groin, through which cannulas are inserted to aspirate subcutaneous fat (**Fig. 1**). However, serious complications have been reported, although they are rare.^2,3)^

**Fig. 1:**
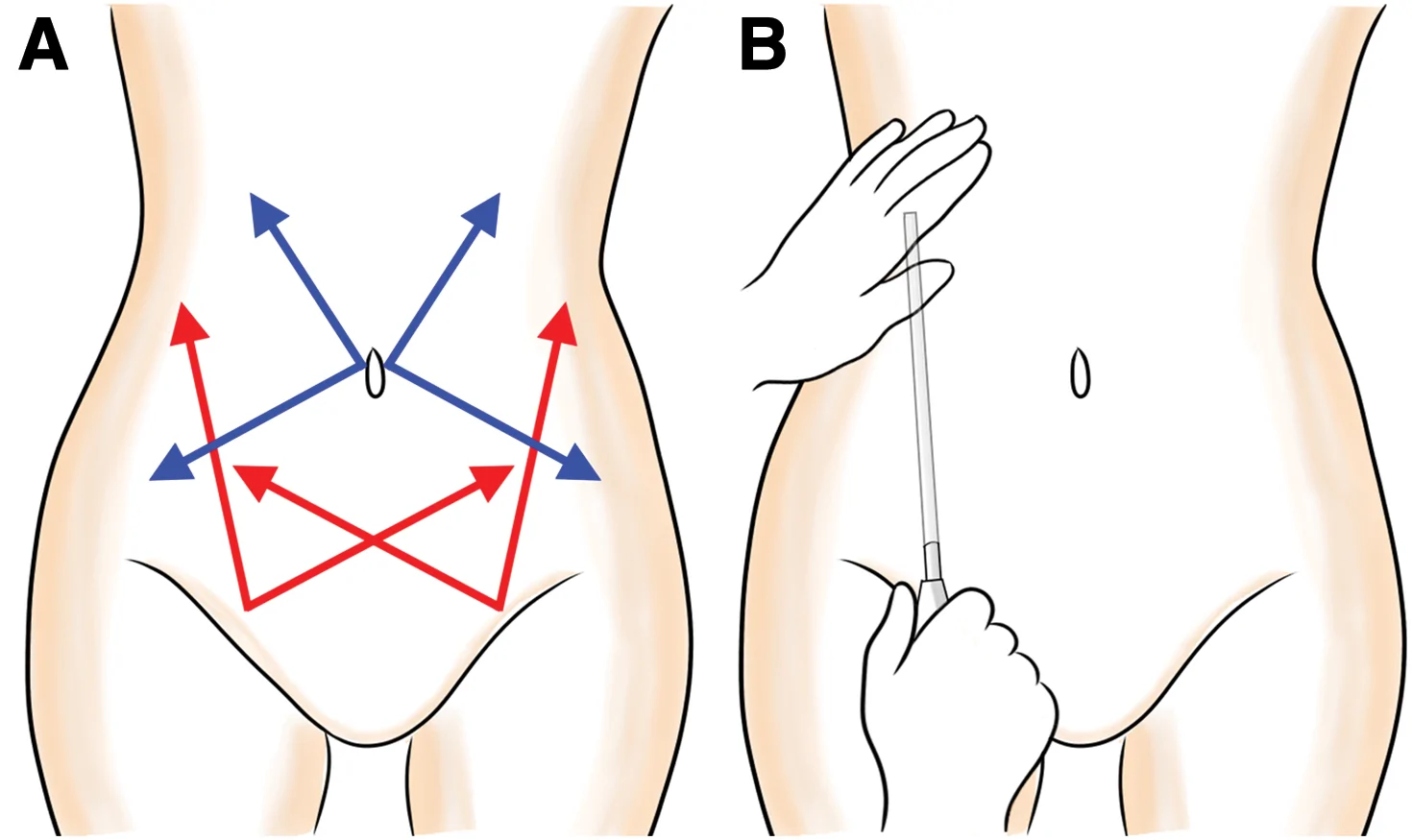
Illustration of liposuction surgery. (**A**) Cannulas are inserted through small incisions at the umbilicus (blue arrows) and inguinal regions (red arrows) in multiple directions to aspirate subcutaneous fat from a wide area. (**B**) Liposuction is performed by moving the cannula back and forth.

We report a case of intra-abdominal abscess formation secondary to small bowel perforation diagnosed 6 days after abdominal liposuction, for which surgical intervention was required. We carefully investigated the origin of the small bowel perforation and considered liposuction the most plausible explanation, although a direct causal relationship could not be definitively established. This case highlights that the diagnosis of bowel perforation may be delayed when no intraoperative abnormalities are recognized during liposuction.

## CASE PRESENTATION

A 55-year-old Japanese woman presented to our hospital with complaints of abdominal pain. The patient had no underlying medical conditions and had undergone 2 previous cesarean sections. She had undergone abdominal liposuction at another institution. Epidural and intravenous anesthesia were administered. Small incisions were made at the umbilicus and bilateral groins (**Fig. 2**), and an US-assisted liposuction system was used. Liposuction was performed using a 3.7-mm US probe and 3.7 and 4.0-mm cannulas. No changes in vital signs or other abnormalities were observed during surgery. Postoperative antibiotics and analgesics were prescribed. During the routine postoperative examination on POD 1, the patient complained of mild abdominal pain and malaise; however, no signs of peritoneal irritation were observed, and the symptoms were attributed to postoperative pain associated with liposuction. On POD 3, the abdominal pain, malaise, and loss of appetite worsened. On POD 4, her temperature was first measured, at 38.0°C. As the symptoms persisted, the patient presented to our hospital on POD 6 (**Fig. 3**). Her BMI was 23 kg/m². Vital signs were as follows: body temperature, 37.9°C; blood pressure, 138/91 mmHg; pulse rate, 95 beats/min; and SpO_2_, 97%. Physical examination revealed mild abdominal distention and tenderness, with rebound tenderness in the left lower quadrant. Laboratory examination revealed a white blood cell count of 11400/μL and a C-reactive protein level of 16.1 mg/dL. Abdominal CT revealed edematous changes in a segment of the small intestine, increased attenuation of the surrounding fat tissue, and an encapsulated fluid collection containing gas around the lesion (**Fig. 4**). No definite evidence of abdominal wall trauma, such as penetrating injury or intramuscular hematoma, was observed. The patient was diagnosed with an intra-abdominal abscess secondary to small bowel perforation, and emergency surgery was performed on the day of admission. A midline abdominal incision was made to enter the abdominal cavity, revealing a small amount of slightly turbid ascites but no obvious peritoneal erythema. The abscess was localized by adhesions between adjacent loops of the small intestine, resulting in a well-encapsulated collection. No fibrinous exudate or purulent exudate was noted beyond the abscess cavity. A single perforation measuring approximately 3 mm was identified in the ileum (**Fig. 5**). No other abnormalities were observed in the small intestine. No foreign bodies, such as fish bones, were observed in the abscess cavity. On examination for signs of abdominal wall trauma, a scar was observed in the peritoneum of the left inguinal region; however, whether it was trauma-related could not be determined. Abscess drainage and partial small bowel resection were performed. Pathological examination revealed ileal perforation, with no other abnormal findings. Granulation tissue formation and marked fibroblast proliferation were observed at the perforation site (**Fig. 6**). The postoperative course was uneventful, and the patient was discharged on POD 7.

**Fig. 2:**
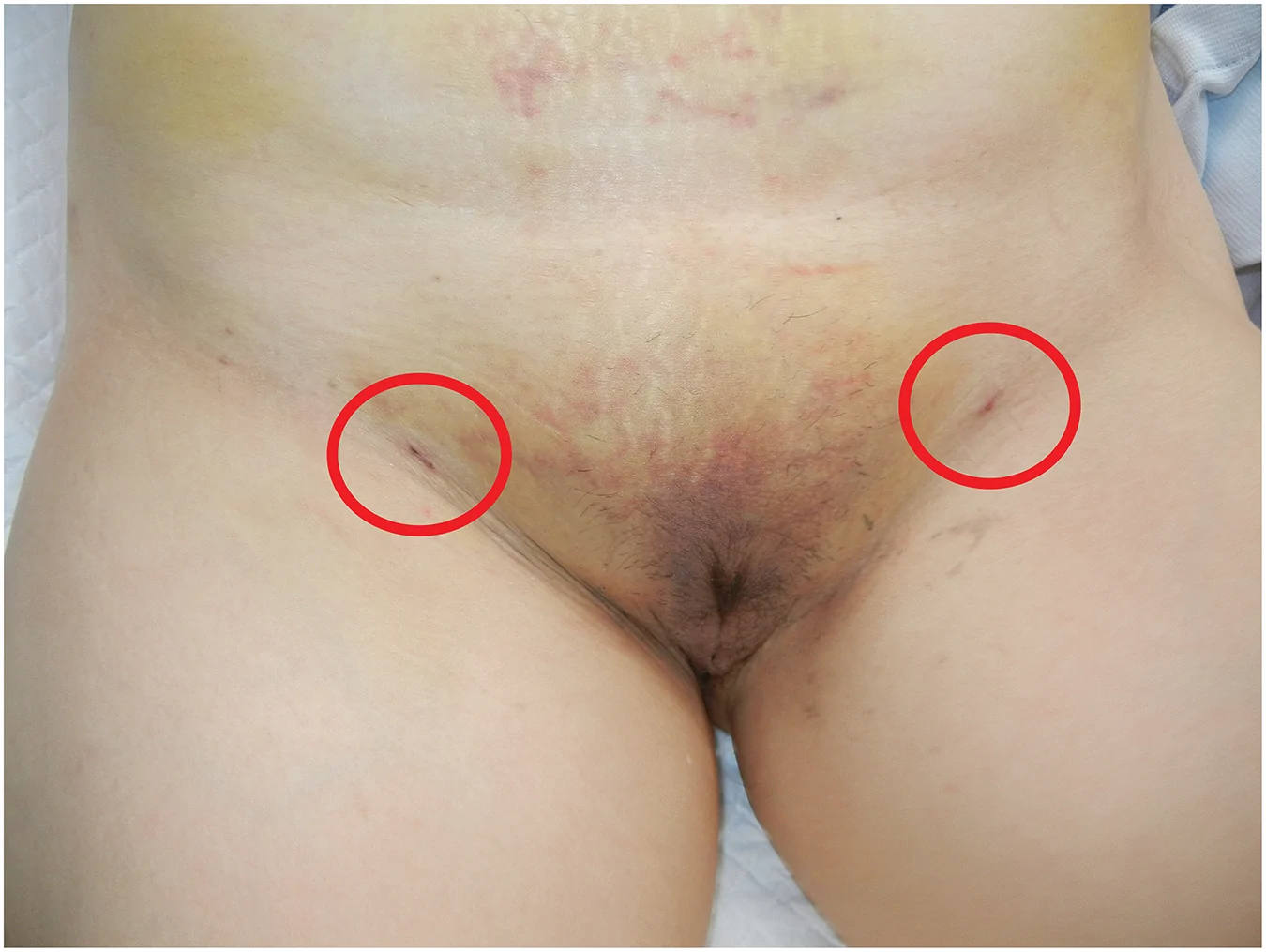
Photograph of the patient. Small incision sites in the bilateral inguinal regions (red ovals). Ecchymosis was observed in the lower abdomen after liposuction.

**Fig. 3:**
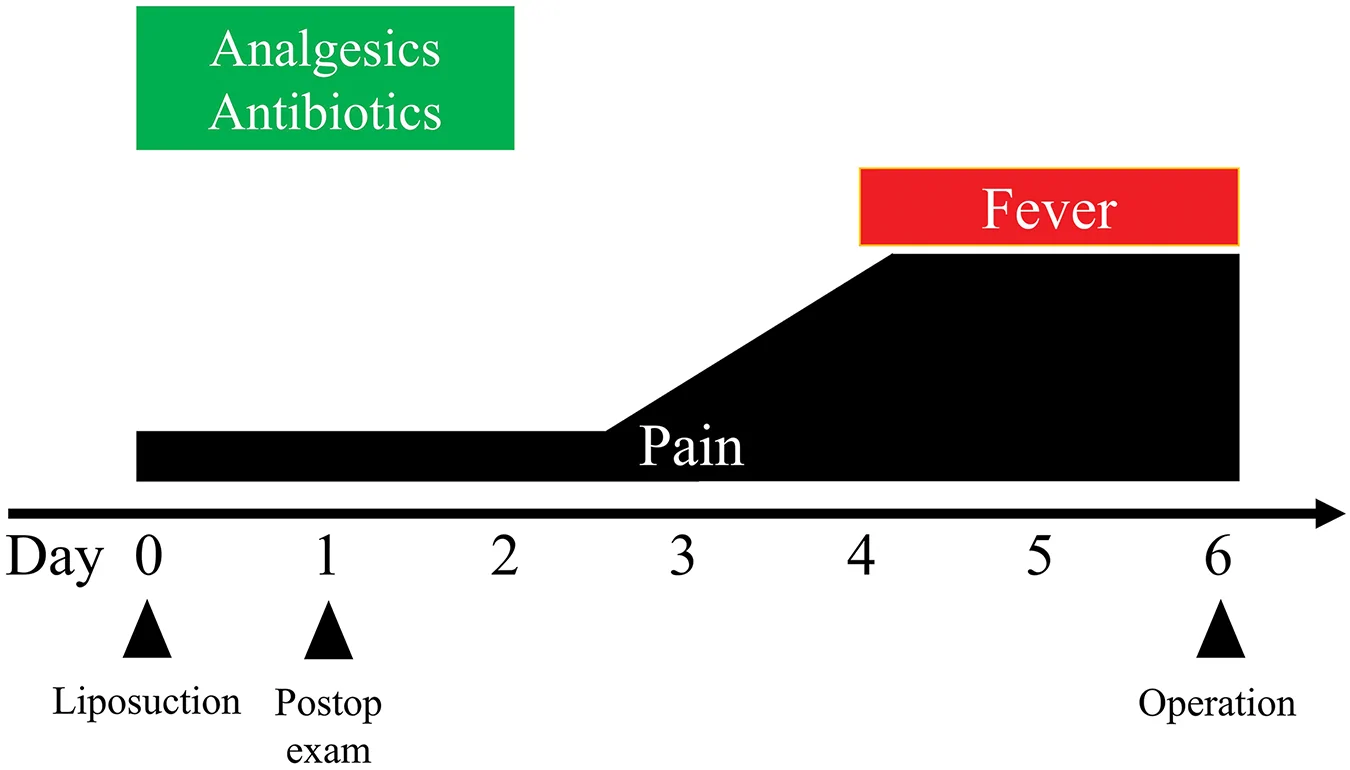
Clinical timeline. Abdominal liposuction was performed on day 0. Mild abdominal pain and malaise were noted on day 1, symptoms worsened on day 3, and fever was first documented on day 4. The patient presented to our hospital on day 6 and underwent emergency surgery.

**Fig. 4:**
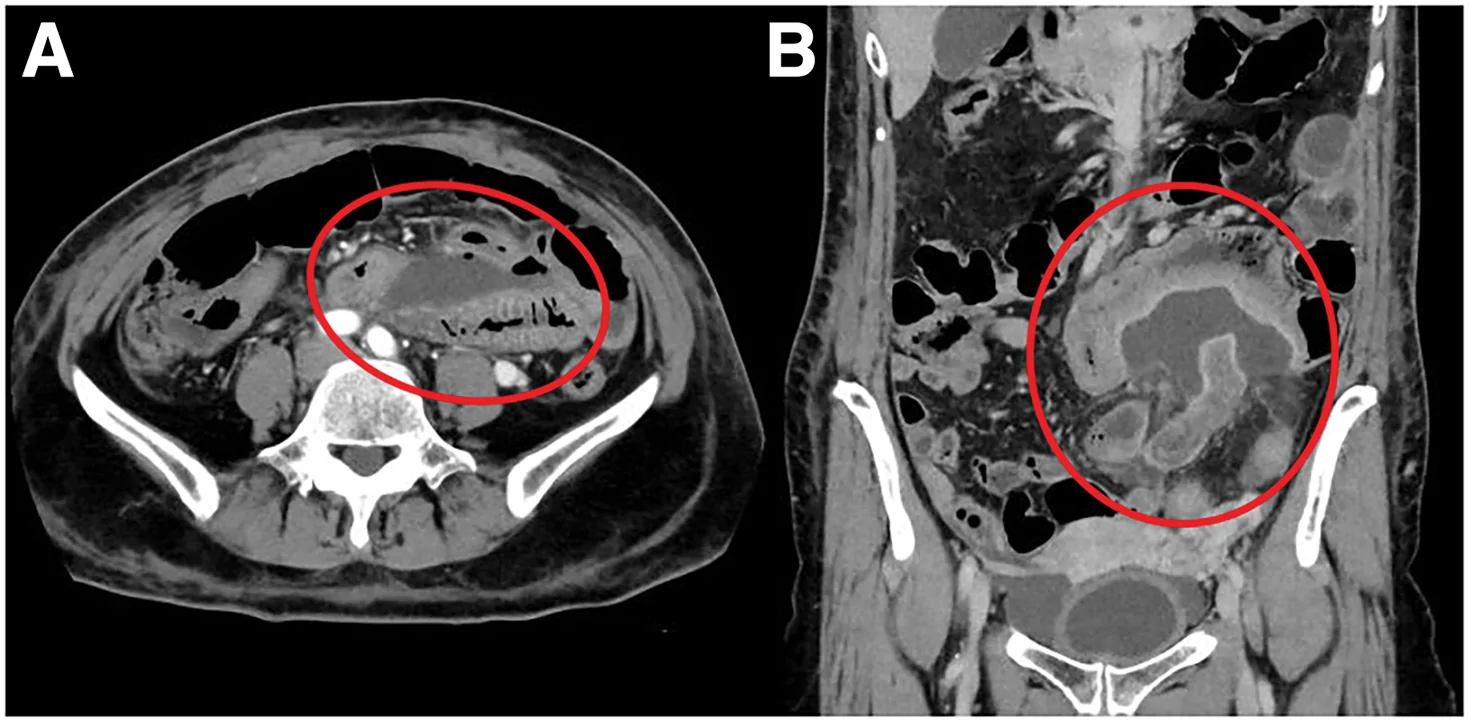
Preoperative contrast-enhanced CT. Edematous changes in a segment of the small intestine and an encapsulated fluid collection containing gas around the lesion are shown (red ovals). Subcutaneous edema and hematoma can be observed in the abdominal wall. (**A**) Axial view. (**B**) Coronal view.

**Fig. 5:**
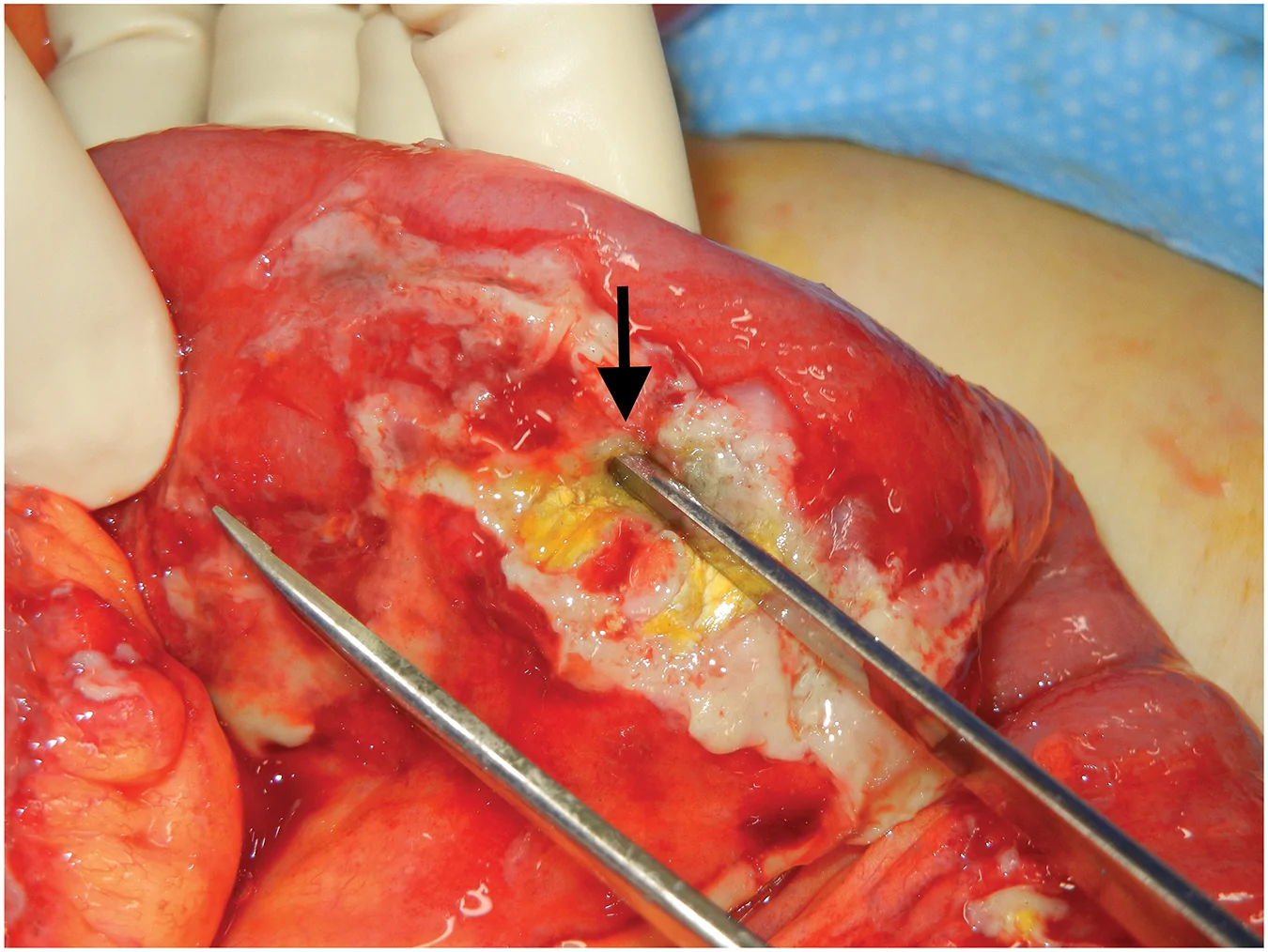
Intraoperative findings. An approximately 3-mm perforation was identified in the ileum (arrow).

**Fig. 6:**
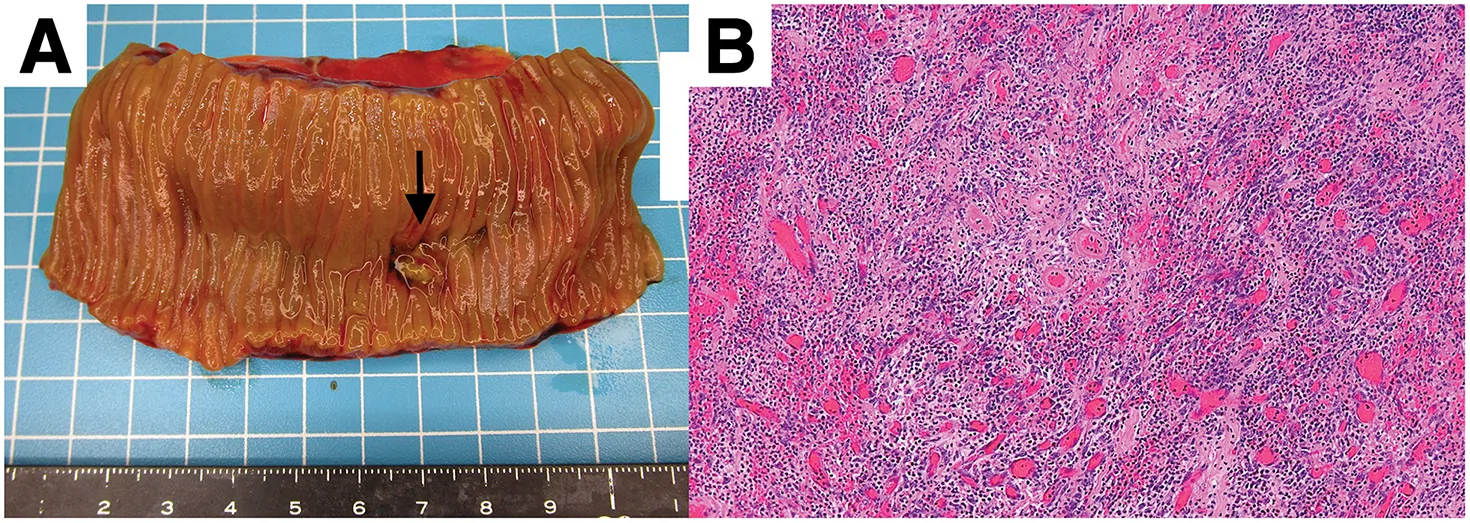
Resected specimen and histopathological findings. (**A**) A perforation can be observed in the resected specimen (arrow), with no other gross abnormalities. (**B**) High-power view showing granulation tissue formation and prominent fibroblast proliferation (hematoxylin and eosin stain).

## DISCUSSION

Several important points emerged from the investigation of the small bowel perforation in this case. First, abdominal liposuction may result in visceral injury. Second, visceral injury may only be diagnosed after a delay, even when no abnormalities are observed during liposuction. In addition, findings suggesting a direct route of injury from the abdominal wall to the injured organ may not be identifiable. In the present case, the cause of the small bowel perforation was carefully investigated. According to the medical history, the patient had not consumed foods containing sharp foreign bodies, such as fish bones, for at least 2 weeks before surgery at our hospital. No foreign bodies causing perforation were identified on CT or during surgery. Moreover, no abnormalities were observed in the small intestine other than the perforated segment, and no abnormal findings other than the perforation site were identified in the resected specimens. Histopathological examination revealed granulation tissue and fibroblast proliferation around the perforation site, suggesting that at least 3–5 days had elapsed since the perforation.^4)^ Based on these findings, liposuction was considered the most plausible explanation for the small bowel perforation, although a direct causal relationship could not be definitively established.

In recent years, the number of cosmetic surgical procedures has increased worldwide, including in Japan.^1)^ In 2024, approximately 380000 cosmetic surgical procedures were performed in Japan, of which 37200 were liposuction procedures.^1)^ Liposuction is generally considered safe, and serious complications are rare. However, reports of severe complications have increased.^2)^ Fatal cases associated with facial liposuction have been reported in Japan.^5)^ Visceral injury during liposuction is rare, and its exact incidence remains unknown.^6)^ Abdominal and visceral injuries during liposuction are the second leading cause of death after pulmonary embolism.^3)^ A PubMed search identified 45 cases of abdominal visceral injury for which sufficient data were available for analysis. These cases, together with the present case, resulted in a total of 46 cases (**Table 1**).^7–32)^ Small bowel injuries, particularly ileal injuries, were the most common, while injuries to the colon, liver, spleen, gallbladder, urinary bladder, and ureter were also reported. Seven of the 46 patients died. Direct penetration by the cannula is generally considered the primary mechanism of visceral injury.^20)^ Indeed, cases in which cannula penetration was suggested based on imaging findings or intraoperative findings have been reported.^21,28)^ Perforation associated with power-assisted liposuction has also been reported.^20)^ However, no reports have clearly demonstrated that ultrasonic vibration or thermal injury was responsible for visceral injury. Regarding negative pressure, one report suggested that strong suction during liposuction may have broken the sutures used for abdominal wall closure^27)^; however, this report also did not state that negative pressure itself directly caused visceral injury. Therefore, in the present case, direct penetration by the cannula may have been involved in the mechanism of visceral injury; however, the possibility that negative pressure, ultrasonic vibration, thermal injury, traction, or other factors contributed to the injury cannot be completely excluded. During liposuction, cannulas are inserted into the subcutaneous tissue and manipulated without direct visualization. Therefore, the abdominal wall may be injured if the insertion angle of the cannula is too steep.^19)^ Reported patient-related risk factors for visceral injury include a history of abdominal surgery, abdominal wall hernia, obesity, rectus diastasis, and other abdominal wall defects.^2,6)^ At least one of these risk factors was reported in 31 of 46 cases (67%). However, because risk-factor information was not available for all cases, the actual prevalence may have been higher. Review of the CT images in the present case revealed no definite fascial defect but did demonstrate rectus diastasis associated with pregnancy (**Fig. 7**). Rectus diastasis is generally defined as a separation of >2 cm.^33)^ In the present case, the maximum distance between the rectus muscles was 2.8 cm, and the patient was diagnosed with rectus diastasis. The supraumbilical region is considered the most vulnerable site because of the weak resistance of the abdominal wall.^34)^ However, a case of small bowel perforation caused by liposuction through an incision placed medial to the anterior superior iliac spine in the groin region has also been reported,^26)^ suggesting that perforation may occur at various sites when vulnerable areas exist in the abdominal wall. In the present case, liposuction was performed through the umbilicus and bilateral groins; however, the insertion site from which the perforation occurred could not be determined. The scar observed in the peritoneum of the left inguinal region was considered potentially related to the previous cesarean sections. In addition, because a midline laparotomy was performed in the present case, even if a small perforation had occurred at the site of rectus diastasis, it may have been transected during the midline incision or overlooked during the intraoperative exploration; therefore, the exact site of perforation could not be determined. Cases in which the site of abdominal wall perforation could not be identified on CT or during surgery have also been reported,^10,22,25)^ suggesting that the site of perforation may not always be visible on preoperative imaging or during intraoperative exploration. Abdominal wall vulnerability due to previous cesarean sections and rectus diastasis may have contributed to the perforation in the small bowel. In the present case, no abnormalities were recognized during the liposuction procedure or on the following day, resulting in a delayed diagnosis of small bowel perforation. After perforation, the intestinal fluid was covered by the surrounding organs before spreading throughout the abdominal cavity, resulting in abscess formation. Because the abscess remained localized, the clinical manifestations were relatively localized, contributing to the delayed diagnosis.

**Table 1:** Demographic and clinical characteristics of reported cases of abdominal visceral injury following liposuction (n = 46)

Characteristic	Value
Age, years	Median 50.5 (range 24–72)
Sex	
Female	37
Male	9
Injured viscus	
Small intestine	34
Ileum	20
Jejunum	5
Small intestine, unspecified	9
Colon	8
Transverse colon	2
Cecum	2
Descending colon	2
Sigmoid colon	1
Colon, unspecified	1
Liver	4
Spleen	3
Gallbladder	1
Urinary bladder	1
Ureter	1
Time from liposuction to diagnosis, days	Median 3 (range 0–8)
Outcome	
Survived	39
Died	7
Risk factors	
Previous abdominal surgery	22
Rectus muscle diastasis	8
Obesity	5
Hernia	4
Abdominal wall weakness	3
None/not described	15

Some patients had multiple injured viscera or risk factors; therefore, these categories were not mutually exclusive.

**Fig. 7:**
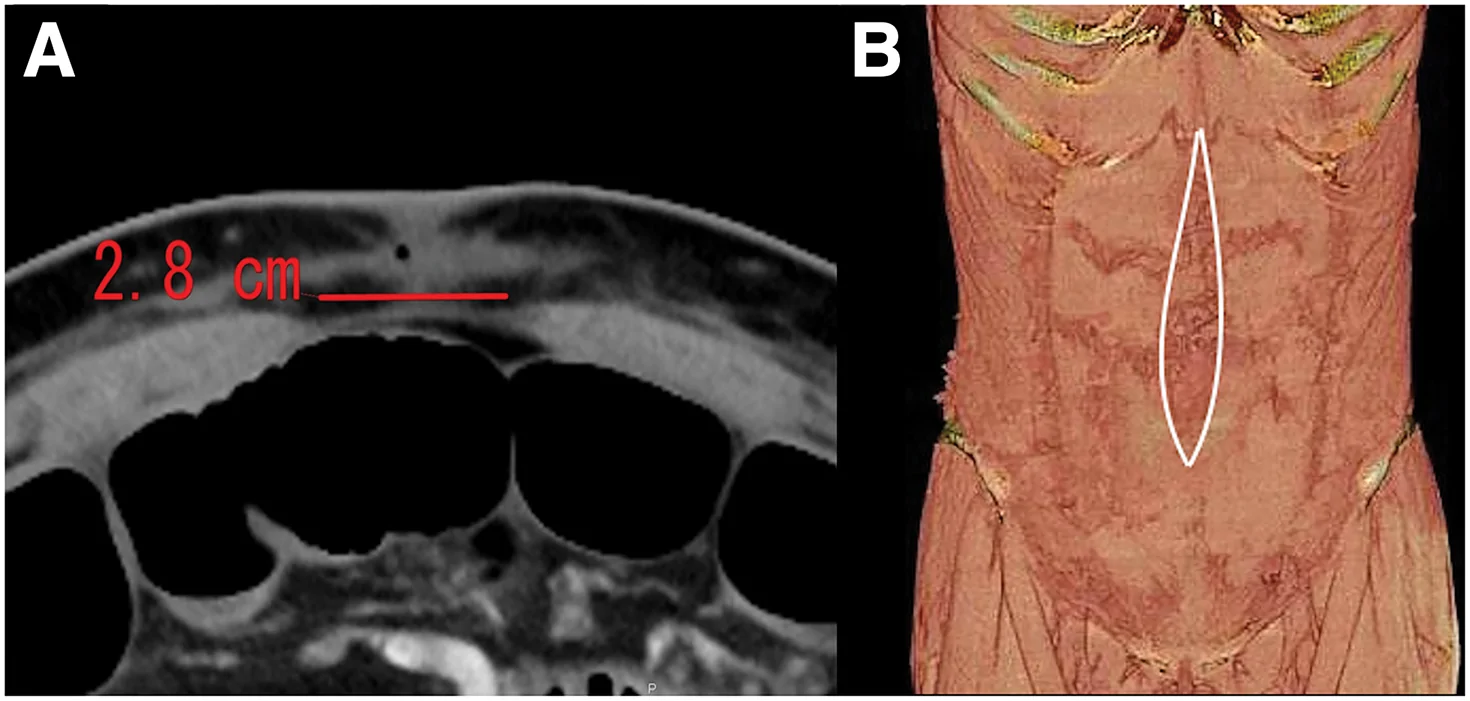
Axial and 3D CT images of the abdominal wall. (**A**) Axial CT image showing a maximum distance of 2.8 cm between the medial borders of the bilateral rectus abdominis muscles, consistent with rectus diastasis. (**B**) 3D CT reconstruction of the abdominal wall. The medial borders of the bilateral rectus abdominis muscles are outlined in white.

In the present case report, the median interval from liposuction to the diagnosis of visceral injury was 3 days (range, 0–8 days). Therefore, visceral injury may not be recognized during liposuction or within the first POD. To prevent visceral injury, detailed preoperative history-taking and abdominal examination are considered valuable for screening. In patients with the aforementioned risk factors, imaging with abdominal ultrasonography or CT is recommended to assess the vulnerability of the abdominal wall.^20,27)^ Regarding the liposuction technique, several measures have been proposed, including avoiding a steep cannula insertion angle or cannula insertion through the umbilicus.^20)^ Additional measures include considering alternative incision sites such as the submammary area, hypochondrium,^20)^ or periareolar region,^27)^ and placing the patient in a hyperextended position.^27)^ One key concern is that the cannula tip cannot be directly visualized by the surgeon during liposuction. In recent years, real-time intraoperative US-guided liposuction has been reported, allowing real-time visualization during the procedure and potentially improving safety.^35)^ Postoperative follow-up is also important, and examination within 24 hours after surgery is recommended.^20)^ It has been shown that rapid diagnosis and early treatment within 72 hours of symptom onset are significantly associated with improved patient outcomes.^2)^ Therefore, even when visceral injury has occurred, patients do not necessarily develop rapid clinical deterioration, and symptoms may progress gradually. Thus, when symptoms persist, even if they are mild, it is important to suspect visceral injury and perform further evaluation.

One limitation of this case is that no clear findings suggestive of cannula penetration through the abdominal wall were identified on preoperative imaging or during intraoperative exploration. Although such findings may have been present but overlooked, a direct causal relationship between liposuction and the small bowel perforation could not be definitively established.

## CONCLUSIONS

Although rare, abdominal liposuction can cause visceral injuries, such as bowel perforation. Depending on the severity of the injury, diagnosis may be delayed, leading to severe and potentially fatal consequences. Surgeons should maintain a high index of suspicion for visceral injuries when evaluating patients with abdominal pain following liposuction.

## DECLARATIONS

### Availability of data and materials

Not applicable, because this is a case report. The clinical information presented in this report is not publicly available, to protect the patient’s privacy.

## References

[1] International Society of Aesthetic Plastic Surgery. ISAPS International Survey on Aesthetic/Cosmetic Procedures Performed in 2024. International Society of Aesthetic Plastic Surgery. 2025. https://www.isaps.org/media/30xldsyf/isaps-global-survey-2024.pdf. Accessed June 1 2026.

[2] Skorochod R, Fteiha B, Gronovich Y. Perforation of abdominal viscera following liposuction: a systemic literature review. Aesthetic Plast Surg 2022; 46: 774–85. 10.1007/s00266-021-02532-9 34462799

[3] Grazer FM, de Jong RH. Fatal outcomes from liposuction: census survey of cosmetic surgeons. Plast Reconstr Surg 2000; 105: 436–46; discussion 447–8. 10.1097/00006534-200001000-00070 10627013

[4] Kumar V, Abbas AK, Aster JC. Robbins basic pathology, 9th ed. Philadelphia: Elsevier/Saunders; 2013. p. 65–8.

[5] Nagano S, Unuma K, Makino Y, et al. Acute upper airway obstruction due to cervical hematoma after cervicofacial liposuction. J Forensic Leg Med 2024; 104: 102697. 10.1016/j.jflm.2024.102697 38772270

[6] Cárdenas-Camarena L, Andrés Gerardo LP, Durán H, et al. Strategies for reducing fatal complications in liposuction. Plast Reconstr Surg Glob Open 2017; 5: e1539. 10.1097/GOX.0000000000001539 PMC5682182 29184746

[7] Ovrebø KK, Grong K, Vindenes H. Small intestinal perforation and peritonitis after abdominal suction lipoplasty. Ann Plast Surg 1997; 38: 642–4. 10.1097/00000637-199706000-00013 9188982

[8] Talmor M, Hoffman LA, Lieberman M. Intestinal perforation after suction lipoplasty: a case report and review of the literature. Ann Plast Surg 1997; 38: 169–72. 10.1097/00000637-199702000-00012 9043587

[9] Sharma D, Dalencourt G, Bitterly T, et al. Small intestinal perforation and necrotizing fasciitis after abdominal liposuction. Aesthetic Plast Surg 2006; 30: 712–6. 10.1007/s00266-006-0078-8 17093873

[10] Mallappa M, Rangaswamy M, Badiuddin MF. Small intestinal perforation and peritonitis after liposuction. Aesthetic Plast Surg 2007; 31: 589–92. 10.1007/s00266-007-0050-2 17659408

[11] Harnett P, Koak Y, Baker D. Splenic trauma during abdominal wall liposuction: a case report. J R Soc Med 2008; 101: 201–3. 10.1258/jrsm.2008.081010 PMC2312382 18387911

[12] King SH, Miller J, Kuan JK. Intraperitoneal bladder rupture after abdominal liposuction. Plast Reconstr Surg 2008; 122: 228e–9e. 10.1097/PRS.0b013e31818d233a 19050506

[13] Kattapuram TM, Avery LL. Ureteral tear at the ureteropelvic junction: a complication of liposuction. Emerg Radiol 2010; 17: 79–82. 10.1007/s10140-009-0797-7 19259711

[14] Raman SR, Pokala N, Cosgrove J, et al. Colocutaneous fistula after suction lipoplasty: case report and literature review. Ann Plast Surg 2010; 64: 503–5. 10.1097/SAP.0b013e3181b4bcaa 20224329

[15] Di Candia M, Malata CM. Aesthetic and functional abdominal wall reconstruction after multiple bowel perforations secondary to liposuction. Aesthetic Plast Surg 2011; 35: 274–7. 10.1007/s00266-010-9591-x 21046103

[16] Coronado-Malagón M, Tauffer-Carrion LT. Jejunal perforation after abdominal liposuction, bilateral breast augmentation and facial fat grafting. Can J Plast Surg 2012; 20: 197–8. 10.1177/229255031202000307 PMC3433819 23997589

[17] Dellière V, Bertheuil N, Harnois Y, et al. Multiple bowel perforation and necrotising fasciitis secondary to abdominal liposuction in a patient with bilateral lumbar hernia. Indian J Plast Surg 2014; 47: 436–40. 10.4103/0970-0358.146650 PMC4292128 25593436

[18] Gialamas E, Oldani G, Modarressi A, et al. Liver trauma during combined liposuction and abdominoplasty: a rare but potentially lethal complication. Aesthet Surg J 2015; 35: NP211–5. 10.1093/asj/sjv028 26254474

[19] You JS, Chung YE, Baek SE, et al. Imaging findings of liposuction with an emphasis on postsurgical complications. Korean J Radiol 2015; 16: 1197–206. 10.3348/kjr.2015.16.6.1197 PMC4644740 26576108

[20] Zakine G, Baruch J, Dardour JC, et al. Perforation of viscera, a dramatic complication of liposuction: a review of 19 cases evaluated by experts in France between 2000 and 2012. Plast Reconstr Surg 2015; 135: 743–50. 10.1097/PRS.0000000000001030 25719693

[21] Ezzeddine H, Husari A, Nassar H, et al. Life threatening complications post-liposuction. Aesthetic Plast Surg 2018; 42: 384–7. 10.1007/s00266-017-1058-x 29387943

[22] Giordano A, Alemanno G, Bici K, et al. A dramatic and rare complication: bowel perforation following abdominal liposuction. G Chir 2019; 40: 429–32. 32003724

[23] Marques Álvarez L, Rodríguez-García R, Palomo Antequera C, et al. Intestinal perforation after liposuction. Cir Esp (Engl Ed) 2019; 97: 536–8. (in Spanish) 10.1016/j.ciresp.2018.12.006 30683272

[24] Singh H, Khazanchi RK, Mahendru S. Successful outcome of post liposuction extensive necrotizing soft tissue infection of abdomen and concomitant jejunal perforation-a case report. Indian J Plast Surg 2019; 52: 238–41. 10.1055/s-0039-1696079 PMC6785307 31602142

[25] De la Fuente JRO, Ferdinand A, Dybas M, et al. Necrotizing soft tissue infection and perforated viscus after suction-assisted lipectomy. Cureus 2020; 12: e8617. 10.7759/cureus.8617 PMC7362623 32676252

[26] Gardener C, Pandis L, Grigatti M, et al. Bowel perforation after liposuction in abdominal contouring surgery: case report. Int J Surg Case Rep 2020; 72: 5–9. 10.1016/j.ijscr.2020.05.012 PMC7276379 32506029

[27] Taha AA, Tahseen H. Spreading awareness: bowel perforation with liposuction. Plast Reconstr Surg Glob Open 2020; 8: e2715. 10.1097/GOX.0000000000002715 PMC7253287 32537364

[28] Pohlan J, Miller H, Lerchbaumer MH, et al. Multiple liver perforations: complication of an outpatient liposuction procedure. Radiol Case Rep 2021; 16: 906–10. 10.1016/j.radcr.2021.01.049 PMC7873377 33603942

[29] Khalaf MH, Sameer M, Khan MB, et al. Abdominal visceral injury, a devastating consequence of abdominal liposuction: a case report and literature review. Cureus 2023; 15: e34378. 10.7759/cureus.34378 PMC9977203 36874694

[30] Bekeny JC, Huffman SS, Thomas C, et al. Small intestinal perforation after 360-degree liposuction: a case report. Aesthetic Plast Surg 2024; 48: 946–52. 10.1007/s00266-023-03567-w 37653177

[31] Jafarian N, Omari RY, Shahbik AE, et al. Iatrogenic liver perforation during liposuction: a case report and in-depth review of clinical presentation, management, and lessons learned. Aesthet Surg J Open Forum 2024; 6: ojad114. 10.1093/asjof/ojad114 PMC10783481 38213469

[32] Darras O, Yacoub S, Mordukhovich I, et al. Successful abdominal wall reconstruction following liposuction catastrophe: a case report and literature review. Ann Plast Surg 2025; 95: 132–7. 10.1097/SAP.0000000000004404 40396685

[33] Reinpold W, Köckerling F, Bittner R, et al. Classification of rectus diastasis-A proposal by the German Hernia Society (DHG) and the International Endohernia Society (IEHS). Front Surg 2019; 6: 1. 10.3389/fsurg.2019.00001 PMC6360174 30746364

[34] Sterodimas A, Boriani F, Magarakis E, et al. Thirtyfour years of liposuction: past, present and future. Eur Rev Med Pharmacol Sci 2012; 16: 393–406. 22530358

[35] Manzaneda Cipriani RM, Duran H, Adrianzen G, et al. Safety and effectiveness of real-time direct vision liposuction performed using intraoperative ultrasonography. Plast Reconstr Surg Glob Open 2025; 13: e6848. 10.1097/GOX.0000000000006848 PMC12150931 40496986

